# Laminin and Integrin in LAMA2-Related Congenital Muscular Dystrophy: From Disease to Therapeutics

**DOI:** 10.3389/fnmol.2020.00001

**Published:** 2020-02-11

**Authors:** Pamela Barraza-Flores, Christina R. Bates, Ariany Oliveira-Santos, Dean J. Burkin

**Affiliations:** Department of Pharmacology, Reno School of Medicine, University of Nevada, Reno, NV, United States

**Keywords:** LAMA2-CMD, Laminin, α7 integrin, muscular dystrophy, muscle, therapeutic

## Abstract

Laminin-α2-related congenital muscular dystrophy (LAMA2-CMD) is a devastating neuromuscular disease caused by mutations in the LAMA2 gene. These mutations result in the complete absence or truncated expression of the laminin-α2 chain. The α2-chain is a major component of the laminin-211 and laminin-221 isoforms, the predominant laminin isoforms in healthy adult skeletal muscle. Mutations in this chain result in progressive skeletal muscle degeneration as early as neonatally. Laminin-211/221 is a ligand for muscle cell receptors integrin-α7β1 and α-dystroglycan. LAMA2 mutations are correlated with integrin-α7β1 disruption in skeletal muscle. In this review, we will summarize laminin-211/221 interactions with integrin-α7β1 in LAMA2-CMD muscle. Additionally, we will summarize recent developments using upregulation of laminin-111 in the sarcolemma of laminin-α2-deficient muscle. We will discuss potential mechanisms of action by which laminin-111 is able to prevent myopathy. These published studies demonstrate that laminin-111 is a disease modifier of LAMA2-CMD through different methods of delivery. Together, these studies show the potential for laminin-111 therapy as a novel paradigm for the treatment of LAMA2-CMD.

## Introduction

Laminin-α2-related congenital muscular dystrophy (LAMA2-CMD), also known as merosin deficient congenital muscular dystrophy type 1A (MDC1A), is a genetic disease caused by mutations in the LAMA2 gene encoding the laminin-α2 protein. Severely affected patients present with neonatal hypotonia and delayed motor milestones. Few children achieve independent ambulation, while most develop respiratory insufficiency, proximal joint contractures, and scoliosis. Patients show elevated serum creatine kinase (CK) and inflammatory cell infiltration in muscle biopsies (Konkay et al., 2016). There is currently no cure or effective treatment for LAMA2-CMD.

**Figure 1:**
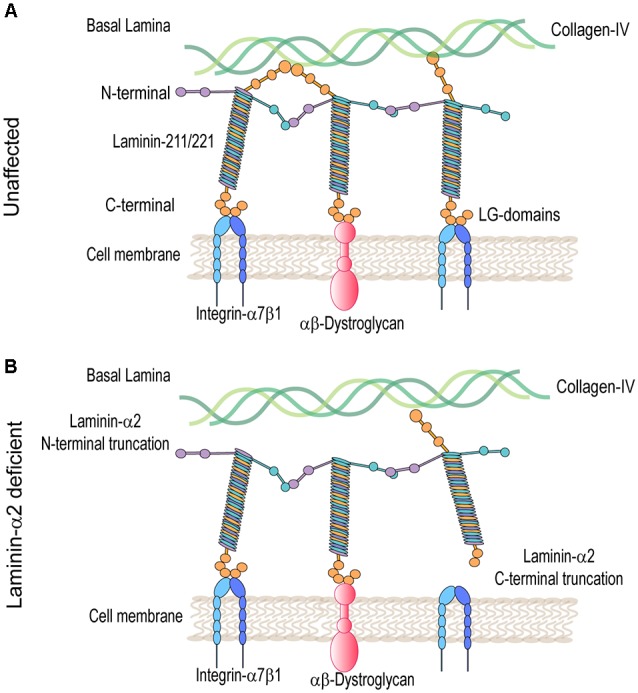
Molecular model in unaffected and laminin-α2-deficient patient muscle. **(A)** Laminin 211/221 heterotrimeric proteins bind through their N-terminal domain to the collagen-IV-rich basal lamina and through their C-terminal domain to muscle cell receptors heterodimers integrin-α7β1 and αβ-dystroglycan protein complexes. **(B)** In LAMA2-CMD, laminins are unable to bind to collagen-IV and/or integrin-α7β1 and α-dystroglycan protein complexes disrupting communication between the basal and muscle cell membrane.

Laminins are ≈900 kDa heterotrimer glycoproteins composed of α, β, and γ chains. They are expressed in several tissues including skeletal muscle. The main isoforms of laminin expressed in healthy adult skeletal muscle are laminin-211 and 221. Laminin-α2 chain is essential for the assembly of these laminins (review on laminin in skeletal muscle; Holmberg and Durbeej, 2013). The laminin-α2 chain is a 380-kDa protein composed of a 300-kDa N-terminal fragment non-covalently bonded to an 80-kDA C-terminal fragment. The N-terminal domain nucleates the association between other laminin-α2 chains and components of the muscle basal lamina including collagen IV and heparan sulfate proteoglycans (Timpl and Brown, 1996). The basal lamina, along with the fibrillar reticular lamina, is a component of the basal membrane or extracellular matrix (ECM; Sanes, 2003). The C-terminal domain is composed of five laminin G (LG) domains, important for cell receptor binding to, predominantly, integrin-α7β1 and the dystroglycan protein complex (DGC; Timpl and Brown, 1996; Figure 1A). These linkages modulate communication between the basal lamina of the ECM and muscle cell cytoskeleton. They also provide mechanical support and stabilization to the sarcolemma during muscle contraction.

Mutations in the LAMA2 gene result in various truncations of the laminin-α2 protein that can result in differential disease presentation and progression. Loss-of-function mutations in the LAMA2 gene are the most common cause of severe LAMA2-related congenital muscular dystrophy (LAMA2-CMD). These are mutations affecting the C-terminal domain, the N-terminal domain, or causing complete ablation of the laminin-α2 protein. Alternatively, variant missense mutations, in-frame deletions, and splice site mutations in the LAMA2 gene often result in a milder, limb-girdle-like, late-onset muscular dystrophy (Geranmayeh et al., 2010; Mohassel et al., 2018). Laminin-α2 deficiency causes disruption of the basal lamina, leading to increased susceptibility to mechanical stress and damage of the myofibers within the muscle. Loss of the laminin-211-rich microenvironment negatively impacts satellite cells (SC) and results in defects in muscle regeneration. Muscle damage results in an inflammatory cell infiltrate leading to replacement of functional skeletal muscle with fibrotic tissue, which exacerbates disease progression (Pegoraro et al., 1996; Nguyen et al., 2019).

Laminin-α2-deficient muscles in both mice and patients exhibit major transcriptome and proteome dysregulation. The major upregulated proteins are components of the ECM and proteins related to muscle regeneration (Taniguchi et al., 2006; van Lunteren et al., 2006; Yanay et al., 2019; Kölbel et al., 2019). The main laminin-α2 cell receptors, α-dystroglycan and integrin-α7β1, are also dysregulated in LAMA2-CMD (Figure 1B). Integrin-α7β1 plays an important role during SC activation, myoblast adhesion, and survival. Consequently, this integrin is important during embryonic development, regeneration, and repair of adult skeletal muscle. Integrin-α7β1 is found to be disrupted in laminin-α2-deficient muscle of multiple mouse models and human biopsies, and therefore, it was studied in LAMA2-CMD. Therapeutic approaches that aim to restore the basement membrane in LAMA2-CMD include LAMA2 gene replacement, engineered linker proteins mini-agrin and αLNND (Reinhard et al., 2017), and laminin-111 treatment. In this review, we will explore laminin-α2 and its receptor integrin-α7 in the LAMA2-CMD disease context, as well as laminin-111 as a disease modifier and therapeutic target for the treatment of LAMA2-CMD.

## Integrin and Laminin Interaction in Laminin-α2-Deficient Skeletal Muscle

**Figure 2:**
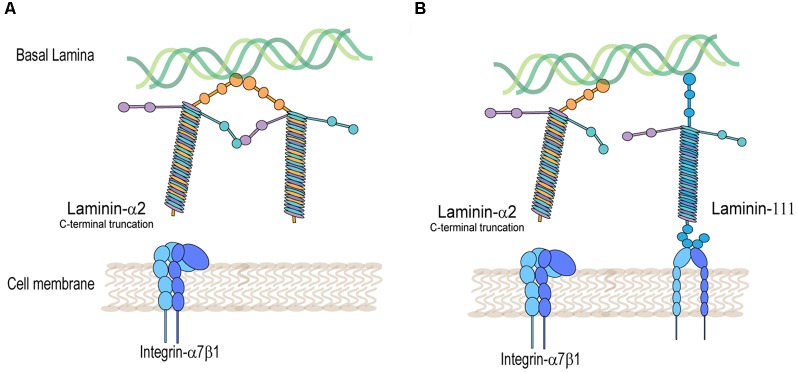
Integrin-α7β1 protein complex is disrupted in laminin-α2-deficient muscle. **(A)** Decreased detection of integrin-α7β1 in the muscle sarcolemma in laminin-α2-truncated muscle. **(B)** Rescued expression of integrin-α7β1 in the muscle sarcolemma by expression of laminin-111 in laminin-α2-deficient muscle.

Integrins are heterodimeric transmembrane glycoprotein receptors made of non-covalently bound α and β subunits (Figure 2). They are essential for cell attachment to the ECM, cell migration, and regulate cellular signal transduction. Integrin clustering and ligand binding to the ECM, including binding to fibronectin, laminins, and collagen, induce conformational changes in integrin, regulating cell adhesion, proliferation, migration, and differentiation (Boppart and Mahmassani, 2019).

Integrin-α7β1 plays a crucial role during embryonic development and in adult skeletal muscle repair by facilitating myoblast adhesion to laminin-111, -211, and -221 (Crawley et al., 1997). Integrin-α7 is encoded by the ITGA7 gene with multiple isoforms produced by developmentally regulated RNA splicing. Alternative RNA splicing of ITGA7 results in integrin-α7A and integrin-α7B cytoplasmic isoforms, as well as integrin-α7X1 and integrin-α7X2 extracellular domain isoforms. The cytoplasmicα7A domain is produced during terminal differentiation of myoblasts and is found in myofibers at the sarcolemma. The cytoplasmic domain α7B-integrin was originally identified during myoblast proliferation (Ziober et al., 1993). The extracellular domain isoform integrin-α7X1 binds strongly to laminin-411, laminin-511, and laminin-521, predominant during muscle embryonic development, while integrin α7X2 binds to laminin-111, expressed during early muscle development. Both integrin α7X1 and α7X2 isoforms bind to laminin-211. This suggests that integrin-α7X1 is involved during early embryogenesis, while integrin-α7X2 is involved at later stages of skeletal muscle development (von der Mark et al., 2002).

The main alternative isoforms for integrin-β1 include integrin β1A and β1D. Whereas integrins β1B and β1C are considered minor isoforms found only in humans, the integrin β1D subunit is expressed in skeletal muscle sarcolemma and associates with integrin-α7 subunit (Belkin et al., 1997).

Laminin-α2 is the main ligand for integrin-α7β1 in skeletal muscle. Integrin-α7 is localized at the neuromuscular and myotendinous junctions and throughout the muscle sarcolemma (Burkin and Kaufman, 1999). Targeted deletion of the integrin-α7 gene in mice results in a mild form of congenital muscular dystrophy and vascular defects (Mayer, 2003; Flintoff-Dye et al., 2005; Rooney et al., 2006). Histopathological studies of this mouse show variation in fiber size, a high percentage of centrally nucleated fibers, elevated CK, mononuclear cell infiltration, and little to no degeneration/regeneration cycles. Expression of laminin-α2 in this mouse, as well as expression of integrin-β1, is unaffected (Mayer et al., 1997). Consistent with this mouse model, patients identified as integrin-α7-negative and laminin-α2-positive present with mild myopathy from birth with no evidence of constituent muscle regeneration (Hayashi et al., 1998). This indicates that other integrin subunits that pair with integrin β1 can partially compensate for the loss of integrin-α7 in muscle.

On the other hand, mutations in the laminin-α2 protein disrupt the expression of integrin-α7β1 in skeletal muscle (Figure 2A). This was shown in several laminin-α2-deficient mouse models and human biopsies of LAMA2-CMD. Using immunofluorescence, studies typically show a decreased detection of integrin-α7B and integrin-β1 subunits in the sarcolemma of laminin-α2-deficient muscle. The dy/dy mouse model of LAMA2-CMD, which has low expression of full-length laminin-α2, exhibits reduced levels of integrin-α7A, integrin-β1D, and irregular integrin-α7B expression (Vachon et al., 1997). Similarly, the dy2J/dy2J mouse model of LAMA2-CMD, which has a mutation in the N-terminal domain, also shows a reduction in integrin-α7A and patchy expression in integrin-α7B (Hodges et al., 1997; Vachon et al., 1997; Cohn et al., 1999). Durbeej (2010) summarizes in a comprehensive review the different mouse models of LAMA2-CMD (Durbeej, 2010).

A case study using biopsies from congenital muscular dystrophy patients with laminin-α2 chain deficiency showed reduced integrin-α7B expression in the sarcolemma of six LAMA2-CMD patients. However, the level of laminin-α2 expression did not correlate with the level of integrin-α7β1 reduction. Restoration of laminin-α2 rescued expression of integrin-α7β1 in the sarcolemma (Vachon et al., 1997). Moreover, the integrin-α7 knockout mouse show no change in laminin-α2 levels (Mayer et al., 1997). Gawlik and Durbeej (2015) reported that loss of integrin-α7 and laminin-α2 in the dy3k/dy3k mouse model, did not result in more severe myopathy. This double knockout mouse presented similar levels of fibrosis, apoptosis, lifespan, and body weight. This same group had previously showed that loss of β-sarcoglycan or dystroglycan in laminin-α2-deficient dy3k/dy3k mice resulted in a dramatically worse muscle disease (Gawlik et al., 2014). This may indicate that the integrin-α7β1 complex has intersecting roles with laminin-α2, while the DGC complex, which includes β-sarcoglycan and dystrophin, may have non-redundant complementary roles. These observations indicate a strong dependence in the expression of integrin-α7β1 receptor to laminin-α2 ligand. This may also indicate that laminin-α2 ligand can compensate for its interaction with secondary integrin complexes.

Doe et al. (2011) showed improvements in myopathy in the dyW mouse model of LAMA2-CMD by transgenically overexpressing integrin-α7. The dyW model presents downregulated and N-terminally truncated levels of laminin-α2 along with severe, early myopathy (Guo et al., 2003). This study showed a threefold increase in lifespan, reduced CLN percent, and reduced macrophage infiltration (Doe et al., 2011). A previous study showed that overexpressing rat integrin-α7 in C2C12 myoblasts promoted adhesion to laminin and overall cell survival, while it did not affect differentiation (Liu et al., 2008). Increased myoblast adhesion to other laminin isoforms may explain the improvements in integrin-α7 overexpressing dyW mouse; however, this remains to be fully explored *in vivo*.

Other integrin dysregulation may drive disease progression in laminin-α2-deficient muscle. Integrin-αV, integrin-α5, integrin-β1, and integrin-β3 were shown to be upregulated in the dyW model of LAMA2-CMD (Accorsi et al., 2015). Integrin-αV was proposed to have a role in TGF-β-mediated fibrosis. More research investigating aberrant disruption of integrin receptors in laminin-α2-deficient skeletal muscle is needed to further our understanding of their roles in LAMA2-CMD disease progression.

## Cell-Based Laminin-111 Treatment of Skeletal Muscle

Skeletal muscle has the capacity to regenerate due to the presence of resident stem cells also known as SCs. These cells are located between the sarcolemma and the basement membrane of muscle fibers (Mauro, 1961). SCs are myogenic progenitors activated in response to muscle injuries and trauma. Upon activation, they proliferate, migrate, and differentiate into myotubes fusing into damaged muscle fibers or generating *de novo* fibers (Morgan and Partridge, 2003; Lepper et al., 2011; Murphy et al., 2011; Sambasivan et al., 2011). There are two types of SC division: apical and symmetrical. Apical or asymmetric division gives rise to two daughter cells of which one is located basally with respect to the other cell, while undergoing differentiation. Symmetrical division, on the other hand, will give rise to two daughter cells that undergo quiescence and maintain the SC pool (Yin et al., 2013).

The ECM, while essential for maintaining muscle fiber integrity (Grounds et al., 2005), also has a crucial function in supporting SC proliferation, migration, and differentiation (Öcalan et al., 1988; Sanes, 2003; Silva Garcia et al., 2019). Disruption of the ECM surrounding SCs, also known as the SC niche, has negative impact on SCs that can lead to failed myogenesis and impaired muscle regeneration (Thomas et al., 2015). The major protein components of the ECM in this niche include collagen (Gillies and Lieber, 2011) and laminin (Yurchenco and Patton, 2009). Laminin-211 and laminin-221, previously known as merosin and merosin-S, are the most abundant laminin isoforms in the adult skeletal muscle (Nissinen et al., 1991; Sasaki et al., 2002). Disrupted levels of laminin-α2 in LAMA2-CMD compromises the integrity of the SC niche, affecting the overall regenerative capacity of the skeletal muscle (Mohassel et al., 2018; Yurchenco et al., 2018). An *in vitro* study by Summers and Parsons (1981) showed that SCs isolated from the dy/dy mouse model of LAMA2-CMD, presented an 80% reduction in muscle colony formation compared to the wild-type controls at 5 months of age. This defect was not shown neonatally or at 1 week of age.

Another *in vitro* study using LAMA2-deficient embryonic stem cells showed that these cells had no defect in differentiating into cardiomyocytes, smooth muscle, and myotubes compared to the wild type. However, the myotubes formed were unstable, detached, collapsed, and degenerated (Kuang et al., 1998a). They found no proliferation or differentiation defects but potential ineffective/abortive development of myotubes (Summers and Parsons, 1981). This points at the role for laminin-α2 in the maintenance of fully differentiated and mature myofibers, essential for myotube stability and survival. An *in vivo* study in mice showed that homozygous LacZ insertion in the LAMA2 gene, effectively disrupting LAMA2 expression, results in incomplete muscle repair and presence of immature myofibers. This study also showed that the early expression of laminin-α2 is important in early embryonic and adult myogenesis (Kuang et al., 1999). Treatment with laminin-α2 protein rescued the instability of LAMA2-CMD myotubes isolated from patient samples (Vachon et al., 1996). An *in vivo* study found that laminin-α2 expression increased levels of SCs in dystrophic gastrocnemius and triceps of dy/dy mice. This provided evidence on the importance of laminin-α2 in the process of skeletal muscle early myogenesis and regeneration of adult muscle. LAMA2 gene replacement in dyW/dyW and dy2J/dy2J mice was capable of restoring the overall health and lifespan of these mouse models of LAMA2-CMD and could serve as a potential therapeutic target (Kuang et al., 1998b). However, immune response against exogenous and now foreign laminin-α2 protein in LAMA2-CMD could impair the benefits of this therapy and exacerbate disease severity.

To address this issue, other laminin isoforms are the target of interest for the treatment of LAMA2-CMD. Laminin-α4 and laminin-α5 are present and upregulated in laminin-α2-deficient muscle (Patton et al., 1997; Kölbel et al., 2019). These laminin isoforms are involved in myogenesis. However, their increased expression in laminin-α2-deficient muscle does not fully rescue its regenerative capacity. Moreover, upon SC activation, an increase in laminin-α4 and laminin-α5 expression is detected, followed by a transient deposition of laminin-α1 in the SC niche (Ishii et al., 2018; Rayagiri et al., 2018). Loss of laminin-α1 impairs SC proliferation and self-renewal leading to defective muscle regeneration indicating its role in SC cycle. Laminin-α1 effects on SC are mediated through integrin-α6 cell receptor and promote SC apical cell division (Rayagiri et al., 2018).

Laminin-α1 contains the highest amino acid homology to laminin-α2 compared to all other laminin chains, and therefore, it is a therapeutic target for LAMA2-CMD. The Laminin-111 heterotrimer differs only in homologous α1 subunit compared to laminin-211. Laminin-111 can be purified from the Engelbreth–Holm–Swarm (EHS) murine sarcoma cell line, where it was first discovered. Several groups reported that *in vitro* treatment with EHS laminin-111 promotes myoblast survival while promoting proliferation and migration (Silva-Barbosa et al., 2008; Goudenege et al., 2010). In a biomaterial study, it was shown that increasing concentrations of laminin-111 integrated into fibrin hydrogels presented a dose-dependent myogenic marker effect. C2C12 myoblasts seeded onto fibrin hydrogel conjugated with a low concentration of laminin-111 showed an increase in myogenin transcription factor, promoting differentiation. At a ninefold increased concentration of laminin-111–fibrin hydrogel, C2C12s showed an increase in MyoD expression, promoting proliferation. This same study reported a dose-dependent increase in VEGF and decrease in IL-6 cytokine secretions effect after 4 days post-seeding onto laminin-111-supported fibrin gels (Marcinczyk et al., 2017). Another study developed hydrogels composed of poly-ethyl glycol conjugated with diacrylate (PEGDA) with and without 5% and 10% laminin-111 (PEGLM). The 5% PEGLM hydrogel showed a more porous structure compared to the other gels, indicating a more ECM-like structure. C2C12s were also seeded in each condition showing flat and spread out morphology in the 5% PEGLM ideal for myofiber formation, compared to rounded, multicellular clusters found in the other hydrogel conditions. A 5% PEGLM C2C12s showed a significant increase in EGF and IL-6 secretion as well as myogenin transcription factor (Ziemkiewicz et al., 2018).

Laminin-111–hydrogel studies show the potential for this biological to affect ECM morphology, alter myoblast differentiation and myokine secretion. Consistent with these studies, SCs expanded *in vitro* on laminin-111–hydrogel were later engrafted onto *mdx* mouse model of Duchenne muscular dystrophy (DMD) and more efficiently regenerated into the dystrophic muscle compared to cells expanded without laminin-111 (Ross et al., 2012). Further studies are necessary to characterize the SC niche and its role in normal muscle regeneration and in the context of LAMA2-CMD. These studies indicate the potential for laminin-111 to structurally support myogenic activity, activate cellular signaling pathways to promote survival, enhance SC engraftment, and promote regeneration in laminin-α2-deficient skeletal muscle.

## Laminin-111 Protein Therapeutics in LAMA2-CMD Mouse Models

Several groups tested the hypothesis that upregulation of endogenous laminin-α1 or exogenous treatment with laminin-111 protein can alleviate disease progression in mouse models of DMD and LAMA2-CMD (Table 1). A study by Rooney et al. (2009) treated the *mdx* mouse model of DMD with weekly intraperitoneal doses of EHS laminin-111. Their results showed an increase in protein levels of integrin-α7 in mice and human DMD myoblasts. They also showed reduced CK, Evans Blue dye-positive fibers, and centrally located nuclei fibers, indicating an increase in sarcolemma stability in dystrophin-deficient muscle (Rooney et al., 2009). These studies were followed by intramuscular treatments with EHS laminin-111 in the golden retriever muscular dystrophy (GRMD) dog model of DMD. The GRMD dog model more closely recapitulates disease progression in DMD compared to the *mdx* mouse model (Kornegay, 2017). These studies showed an increase in muscle regeneration and repair and *in vivo* force measurements in the dog’s hindlimbs (Barraza-Flores et al., 2019).

**Table 1:** Laminin-111 therapy articles in muscular dystrophy animal models.

References	Method	Model	MD*	Results
Gawlik et al. (2004)	Transgenic over expression of laminin-α1	Dy^3K^ mouse model	LAMA2-CMD	- Laminin-α1 expressed in skeletal muscle - Rescued myopathy - Decreased CLN, increased survival and weights
Gawlik et al. (2006a)	Transgenic over expression of laminin-α1	Dy^3K^ mouse model	LAMA2-CMD	- Laminin-α1 expressed in peripheral nerves - Delayed myopathy - Restored peripheral neuropathy
Gawlik et al. (2006b)	Transgenic over expression of laminin-α1	Dy^3K^ mouse model	LAMA2-CMD	- Translocation of integrin α7β1 protein complex to the sarcolemma in LAMA1-overexpressing mice
Rooney et al. (2009)	i.p. EHS laminin 111	Mdx mouse model	DMD	- Increased sarcolemma stability - Increased integrin α7β1 protein complex
Gawlik and Durbeej (2010)	Transgenic overexpression of laminin-α1	Dy^3K^ mouse model	LAMA2-CMD	- 1.5- to 2-year old LAMA1 overexpressing mice shows decreased myopathy - Small differences in weights, fibrosis, CK levels compared to wild-type controls
Gawlik et al. (2011)	Transgenic overexpression of laminin-α1	Mdx mouse model	DMD	- No changes in CLN, fiber size, sarcolemma damage, grip strength, CK - Increased integrin α7β1 protein complex
Rooney et al. (2012)	i.p. EHS laminin 111	dy^W^ mouse model	LAMA2-CMD	- Increased survival - Decreased fibrosis, immune infiltration, and CLN
Ross et al. (2012)	Laminin-111-treated myoblast engraftment	Mdx	DMD	- Myoblasts expanded on laminin-111 contributed more to muscle regeneration in *mdx* mouse model compared to fibronectin-expanded cells
van Ry et al. (2014)	i.m. EHS laminin 111	dy^W^ mouse model	LAMA2-CMD	- Laminin-111 restores muscle regeneration in cardiotoxin-treated muscle
Perrin et al. (2017)	CRISPR/dCas9 overexpression of laminin-α1	C2C12 myoblasts and Mdx mouse model	DMD	- Laminin-α1 expression in C2C12 and *mdx* mouse model
Gawlik et al. (2018)	Transgenic overexpression of laminin-α1	Dy^2J^	LAMA2-CMD	- Decreased immune cell infiltration, fibrosis - Increased activity and female weights
Barraza-Flores et al. (2019)	i.m. EHS laminin 111	GRMD dog model	DMD	- Increased regeneration, *in vivo* force measurements, muscle weight - Decreased CLN
Kemaladewi et al. (2019)	CRISPR/dCas9 overexpression of laminin-α1	Dy2J mouse model	LAMA2-CMD	- Laminin-α1 expression in skeletal muscle - Increased functional and strength measurements - Improved nerve conduction

Note. Articles are in chronological order indicating the animal model, related human disease, and a summary of most impactful results. *MD, muscular dystrophy; LAMA2-CMD, laminin-α2-related congenital muscular dystrophy; DMD, Duchenne muscular dystrophy; GRMD, golden retriever muscular dystrophy; EHS, Engelbreth–Holm–Swarm; CLN, centrally located nuclei; CK, creatine kinase; CRISPR, clustered regularly interspaced short palindromic repeats.

Similarly, Rooney et al. (2012) treated the dyW mouse model of LAMA2-CMD, with weekly intraperitoneal injections of EHS laminin-111. These studies reported an increase in life-span and a decrease in centrally located nuclei fibers, fibrosis, and immune infiltration. In a subsequent study in dyW mouse, intramuscular injections with EHS laminin-111 showed improvements in muscle repair post cardiotoxin treatment of hindlimbs. Treated muscles showed increased integrin-α7β1 complex, increased myofiber size, and number (van Ry et al., 2014).

Other groups explored the transgenic overexpression of laminin-α1 in several mouse models of LAMA2-CMD and DMD. Overexpression of laminin-α1 results in proper assembly of laminin-111 and translocation to the sarcolemma as demonstrated by several transgenic mice using immunofluorescence. Gawlik et al. (2011) showed that the transgenic overexpression of laminin-α1 in the *mdx* mouse model of DMD, while increasing levels of integrin-α7β1, had little to no effect in myopathy. This could mean that the presence of endogenous laminin-211/221 in the *mdx* mice may reduce the efficacy of laminin-α1 transgenic expression. These studies may also indicate that in dystrophin-deficient muscle, exogenous treatment can repair sarcolemmal integrity, perhaps through signaling stimulated exclusively through an external laminin-111 receptor binding.

Transgenic overexpression of laminin-α1 was also achieved in two different models of LAMA2-CMD. Transgenic expression of laminin-α1 in dy3K mice showed a drastic increase in survival, weights, decreased centrally located nuclei, and alleviated myopathy (Gawlik et al., 2004). This model was also used to investigate the role integrin-α7β1 plays in laminin-α1 treatment of LAMA2-CMD. They found that the non-transgenic dy3K mouse already shows significantly increased synthesis of integrin-α7β1 (via transcript and protein levels) compared to the wild type. However, the integrin-α7 subunit increase was not detected in the skeletal muscle sarcolemma, indicating that it was not translocated from the cytoplasm to the sarcolemma. Interestingly, when laminin-α1 was transgenically overexpressed, integrin-α7 was detected in the sarcolemma of the muscle (Gawlik et al., 2006b) perhaps due to available ligand-binding receptors (Figure 2B). This provides a potential mechanism of action in which overexpression of integrin-α7 is able to prevent muscle disease in LAMA2-CMD. Additionally, aged dy3K mice overexpressing laminin-α1 had a lifespan of up to 1.5–2 years of age, and all non-transgenic dy3K mice died. These older laminin-α1 transgenic dy3K mice had improved survival; however, they had significantly lower weights, muscle strength, and exhibited muscle fibrosis compared to the wild type (Gawlik and Durbeej, 2010).

A new transgenic mouse able to express laminin-α1 in the peripheral nervous system of the dy3K mouse was reported. Results showed rescued peripheral neuropathy such as improved Schwann cell basement membrane and nerve conduction (Gawlik et al., 2006a). In order to show that laminin-111 is a disease modifier of LAMA2-CMD, laminin-α1 was overexpressed in a model with a different LAMA2 mutation, the dy2J. These studies provided a comparison between the most severe, laminin-α2 negative, and the less severe, laminin-α2-reduced mouse models. Transgenic dy2J mouse also showed laminin-α1 expression in the heart, skeletal muscle, and sciatic nerve. Functional assessments showed increased grip strength and activity, while histopathology revealed reduced central nucleation and fibrosis (Gawlik et al., 2018). Together, these studies indicate that laminin-111 can modify muscle disease progression in LAMA2-CMD independent of LAMA2 mutation and is a therapeutic target for LAMA2-CMD.

Ubiquitous expression of laminin-α1 in dystrophic skeletal muscle could be achieved through gene replacement using adeno-associated virus (AAV) delivery. However, this approach is currently limited by the ~10-kb size of the LAMA1 and LAMA2 cDNAs. Production of micro-laminin-α2 retains the activity if the full protein is a possibility, but challenges include producing constructs that successfully associate with laminin-β1, laminin-β2, and laminin-γ1 chains and are functional.

Recent advancements in CRISPR/Cas9 technology showed the capacity for catalytically inactive dCas9 (or dead Cas9) nuclease to upregulate genes of interest by activating to gene promoters. A recent study in the dy2J mouse model used the *Streptococcus pyogenes* dead Cas9 (sadCas9) and fused it to eight copies of the transcriptional activator VP16. Five guide RNAs (gRNAs) were designed to target upstream LAMA1 promoter. CRISPR and VP64–sadCas9 along with gRNAs were packaged into AAVs and delivered *in vitro*. A combination of gRNAs was found optimal for the upregulation of LAMA1 and tested *in vivo*. Laminin-α1 was successfully upregulated in the skeletal muscle and sciatic nerve of dy2J mice *via* LAMA1 promoter activation. Histopathology and functional studies showed reduced fibrosis, increased fiber diameters, and myelination of sciatic nerves, as well as improvements in activity, force measurements, and nerve conduction after a single injection of AAV-packaged CRISPR/VP64–sadCas9 (Kemaladewi et al., 2019).

Potential problems of this approach include off-target activities, regulating CRISPR/VP64–sadCas9 activity so laminin-α1 expression is more precisely controlled, assessing the long-term impacts of CRISPR/Cas9 system in human muscle, potential requirement for multiple dosing, and immune response to the AAV delivery system. Nonetheless, once these limitations are addressed, this approach represents an exciting new frontier for the treatment of LAMA2-CMD.

## Future Perspectives

Laminin-211/221 is the main ligand for muscle cell receptors: the dystroglycan complex (DGC) and integrin-α7β1 heterocomplex. Therefore, mutations in laminin-α2 lead to disrupted communication between the basal lamina and muscle cell receptors. These interactions are detrimental for mechanical support during muscle contraction. Overexpression of integrin-α7 in a LAMA2-CMD mouse model was reported to reduce muscle disease progression in a mouse model of LAMA2-CMD. The observations indicate that overexpression of integrin-α7 can reestablish the linkage between muscle cells and laminin isoforms in laminin-211/221-deficient muscle. Possible approaches to restore integrin-α7β1 to the sarcolemma of LAMA2-CMD patients include AAV–ITGA7 expression. These studies were performed in two models of DMD and shown to improve muscle pathology and physiology (Heller et al., 2013, 2015). Problems with an AAV–ITGA7 approach include regulating ITGA7 expression in muscle and immune response to AAV delivery systems. Another approach is using small molecules that target an increase in integrin-α7. A muscle cell-based screen has recently identified SU9516 and a structural analog Sunitinib (an FDA-approved compound), which increase the α7β1 integrin in the muscle of *mdx* mice (Sarathy et al., 2017; Fontelonga et al., 2019). These small molecules could be used to restore the integrin-α7β1 linkage system to the sarcolemma and reduce muscle disease progression in LAMA2-CMD. However, this approach remains to be explored.

Laminin-111 was shown to be an effective protein replacement therapy for LAMA2-CMD by multiple groups (Table 1). EHS-derived laminin-111 was studied in multiple animal models with different forms of muscular dystrophy. Treatment with laminin-111 in LAMA2-CMD mouse model increased life expectancy, muscle function, and muscle regeneration and repair. Additionally, laminin-111 protein therapy may provide the unique ability to deliver different concentrations of the biological throughout the body. High local concentration of laminin-111 may enhance myoblast differentiation while maintaining their survival through myokine signaling. Low local laminin-111 concentrations may induce myogenic proliferation. However, the effects of laminin-111 on SCs and myogenic proliferation and differentiation require further investigation.

Translation of laminin-111 protein therapy from the bench to the bedside will require production of recombinant human laminin-111 protein. Human laminin-111 has 70% amino acid homology compared to the mouse isoform. Cross-species differences in amino acid sequence and/or glycosylation patterns may trigger an immune response in mice or humans. Treatment using EHS mouse laminin-111 in the GRMD dog model did stimulate an immune response, but appeared not to have any adverse effects in this large animal model of DMD. Potential problems for laminin-111 therapeutics include: (1) production of a 900-kDa recombinant human laminin-111 in sufficient quantity and purity to treat LAMA2-CMD patients; (2) potential immune response since multiple injections will be required throughout the lifespan of the patient; (3) care in administering the recombinant laminin to ensure it does not polymerize during intravenous treatments; and (4) determining dosing requirements and drug efficacy. Once these problems are resolved, then laminin-111 could serve as a potent therapy for LAMA2-CMD and other muscular dystrophies.

Finally, another approach to produce laminin-111 in skeletal muscle is *via* endogenous upregulation of laminin-α1 protein chain. Since the LAMA1 cDNA is ~10 kb, then delivery using AAV is not feasible. A potential solution may be upregulation *via* LAMA1 promoter stimulation using CRISPR/sadCas9–VP64 technology. Treatment with CRISPR/sadCas9–VP64 in the more severe laminin-α2 negative mouse model, the dy3k, is needed to assess the efficacy of this approach with congenital onset. Although CRISPR/sadCas9–VP64 may be the strongest potential approach to upregulate laminin-α1 in patients, delivery challenges may include immune response against AAV.

In the development of several approaches based on integrin-α7β1 regulation, laminin- and cell-based therapies, laminin-111 protein replacement therapy, or laminin-α1 enhancement, each represents novel approaches for LAMA2-CMD. Each approach has unique strengths and weaknesses that will require extensive testing and assessment before entering clinical trials. Finally, a combinatorial approach using each of these approaches may serve as the most effective approach for treating this devastating muscle disease.

## Author Contributions

PB-F conceptualized and designed the article. PB-F, CB, and AO-S drafted the article. DB critically revised the article.

## Conflict of Interest

The University of Nevada, Reno, has a patent on the therapeutic use of laminin-111 and its derivatives. This patent has been licensed to Prothelia Inc., and the University of Nevada, Reno has a small equity share in this company.
